# Myelination and mTOR

**DOI:** 10.1002/glia.23273

**Published:** 2017-12-06

**Authors:** Gianluca Figlia, Daniel Gerber, Ueli Suter

**Affiliations:** ^1^ Institute of Molecular Health Sciences, Department of Biology Swiss Federal Institute of Technology, ETH Zürich Zürich CH 8093 Switzerland

**Keywords:** metabolism, mTOR, myelin, oligodendrocyte, Schwann cell

## Abstract

Myelinating cells surround axons to accelerate the propagation of action potentials, to support axonal health, and to refine neural circuits. Myelination is metabolically demanding and, consistent with this notion, mTORC1—a signaling hub coordinating cell metabolism—has been implicated as a key signal for myelination. Here, we will discuss metabolic aspects of myelination, illustrate the main metabolic processes regulated by mTORC1, and review advances on the role of mTORC1 in myelination of the central nervous system and the peripheral nervous system. Recent progress has revealed a complex role of mTORC1 in myelinating cells that includes, besides positive regulation of myelin growth, additional critical functions in the stages preceding active myelination. Based on the available evidence, we will also highlight potential nonoverlapping roles between mTORC1 and its known main upstream pathways PI3K‐Akt, Mek‐Erk1/2, and AMPK in myelinating cells. Finally, we will discuss signals that are already known or hypothesized to be responsible for the regulation of mTORC1 activity in myelinating cells.

## Main Points

- Myelination is metabolically demanding.
- The metabolic regulator mTORC1 controls differentiation of myelinating cells and promotes myelin growth.
- mTORC1‐independent targets of the PI3K‐Akt and Mek‐Erk1/2 pathways may also be significant in myelination.

## 1 MYELINATION AND METABOLISM

In the seminal work describing the cells still bearing his name, Theodor Schwann also introduced for the first time the word metabolism to describe “chemical changes either in the component particles of the cell itself, or in the surrounding cytoblastema” (Schwann, Smith, & Schleiden, 1847). Almost two centuries later, it is tempting to see an inadvertent biological meaning in this fortuitous coincidence.

### 1.1 The metabolic challenge of myelination

**Figure 1:**
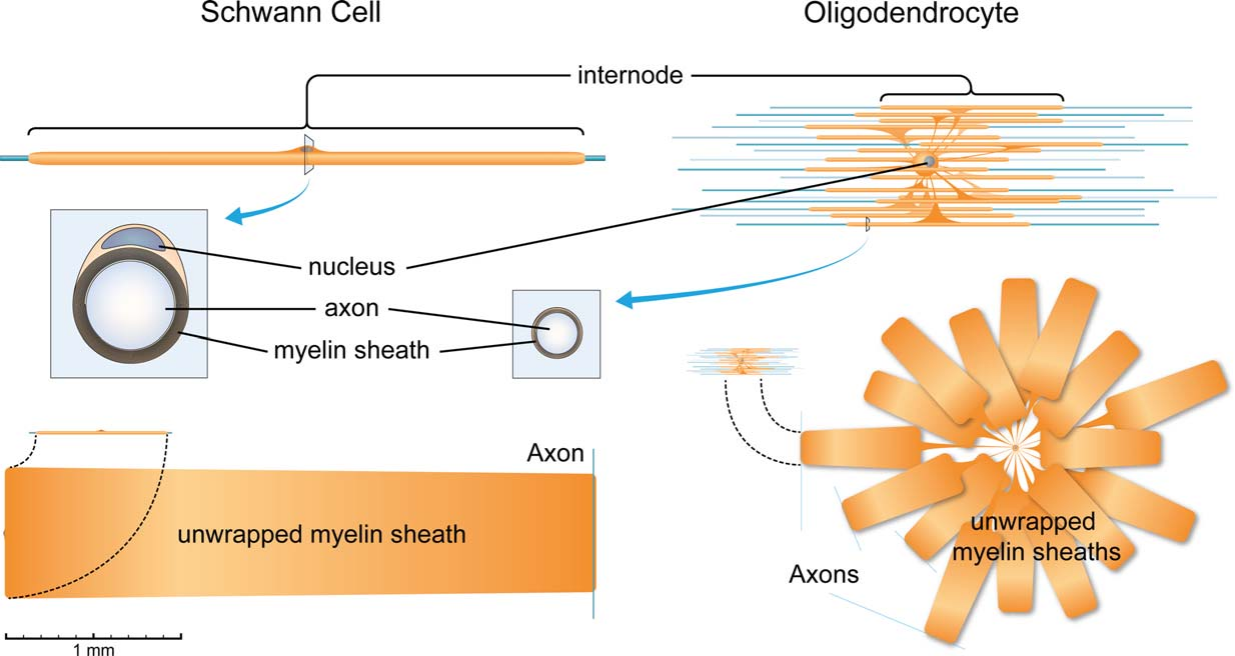
Myelination entails a striking expansion of the cell membrane. Schematic representation of a myelinating SC and a myelinating OL, drawn to scale. To illustrate the extent of membrane expansion during myelination, the corresponding myelin sheaths have been “unwrapped” in the bottom part. The following average dimensions have been considered: Myelinating SCs in the rat sciatic nerve wrap their membrane 72–94 times around axons and outstretch 650‐μm long internodes on average (Webster, 1971); myelinating OLs in the rat optic nerve wrap up to 30 times, extend on average 200‐μm long internodes (Butt & Ransom, 1993; Wiggins, Fuller, Brizzee, Bissel, & Samorajski, 1984), and myelinate on average 16 axons (Butt & Ransom, 1993). Of note, although no data are available for rat OLs, variations in the length of the internodes myelinated by a single OL have been reported in mice (Chong et al., 2012). For SCs and OLs, the total surface area of myelinating cells has been estimated, on average, to 20 × 10^5^ μm^2^ (Pfeiffer, Warrington, & Bansal, 1993; Webster, 1971)

Schwann cells (SCs) in the peripheral nervous system (PNS) and oligodendrocytes (OLs) in the central nervous system (CNS) generate myelin through spiral wrapping of their plasma membranes around axons. This process entails a remarkable expansion of the surface area of myelinating cells (Figure 1), and consequently, a congruous amount of proteins and lipids must reach the nascent myelin sheath. It has been estimated that OLs synthesize 10^5^ proteins per minute during active myelination (Pfeiffer et al., 1993), equal to about 1,500 proteins per second. As a comparison, well‐known “protein factory cells” like plasma B cells are estimated to synthesize 1,500–2,000 antibody molecules per second (Nossal & Makela, 1962). Additionally, several lines of evidence indicate that also most lipids are de novo synthesized by myelinating cells. First, Myrf and Krox20, the master transcription factors for CNS and PNS myelination, respectively, bind to regulatory regions of genes involved in lipid synthesis (Bujalka et al., 2013; Jang et al., 2010). Second, all cholesterol in peripheral nerves and more than 90% of cholesterol in the brain is locally synthesized (Jurevics & Morell, 1995, 1994). Third, no major contribution of exogenous palmitic, stearic, or oleic acid was detected in the developing brain (Edmond, Higa, Korsak, Bergner, & Lee, 1998; Marbois, Ajie, Korsak, Sensharma, & Edmond, 1992). Fourth, myelination was impaired after conditional deletion of enzymes necessary for cholesterol biosynthesis (squalene synthase) or deletion of SREBP‐cleavage activating protein (SCAP) upstream of SREBP transcription factors (see below) in myelinating cells (Saher et al., 2005, 2009; Verheijen et al., 2009). Thus, assuming a protein‐to‐lipid molar ratio in myelin of 1:186 (Gent, Gregson, Gammack, & Raper, 1964; O'Brien & Sampson, 1965), it appears likely that myelinating cells must synthesize several thousands of new lipid molecules per second. However, recent data have revealed that lipid synthesis in astrocytes is also necessary for OL myelination, indicating that some contribution from other cell types in the brain is not to be excluded (Camargo et al., 2017; Schmitt, Castelvetri, & Simons, 2015).

### 1.2 Slowing down, but never at rest

Myelin synthesis continues into adulthood in a temporally and spatially controlled manner (Baumann & Pham‐Dinh, 2001; Jessen, Mirsky, & Lloyd, 2015; Snaidero & Simons, 2017). As most cellular products, also myelin undergoes turnover, although the extent and rate are not completely clear. The use of radioactive precursors to determine the half‐lives of individual myelin components has yielded diverging results, probably due to metabolism and recycling of the radioactive molecule, different routes of administration, and different time intervals between administration of the radioactive molecule and measurements (Benjamins & Smith, 1977). More recently, isotope labeling and mass spectrometry have been employed to overcome these pitfalls. Using a pulse of deuterated water, the half‐lives of myelin lipids were estimated to range from 360 days for cholesterol to 24 days for phosphatidylcholine (Ando, Tanaka, Toyoda, & Kon, 2003). Following an analogous approach in which proteins were pulse‐labeled with ^15^N followed by a chase period with ^14^N, the myelin proteins myelin basic protein (MBP), proteolipid protein (PLP), myelin oligodendrocyte glycoprotein (MOG), and 2′,3′‐cyclic‐nucleotide 3′‐phosphodiesterase (CNP) were identified in an unbiased manner among the long‐lived proteins of the brain (Toyama et al., 2013). Although myelin proteins and lipids are probably overall relatively stable, in the long term they appear to be exchanged substantially according to a study conducted on human postmortem brain samples (Yeung et al., 2014). Of note, studies on myelin turnover in the PNS are rather scarce at this time and require further research attention (Sheean et al., 2014).

Besides replacing “old” myelin, new myelin can also be added to modulate certain neural circuits based on experience. This recently described process has been named adaptive myelination or myelin plasticity, in analogy to synaptic plasticity (Forbes & Gallo, 2017; Mount & Monje, 2017). For example, learning of a complex motor task entails proliferation and differentiation of adult oligodendrocyte progenitor cells (OPCs), and presumably generation of some new myelin segments (McKenzie et al., 2014). However, also apposition of new myelin lamella to already existing internodes might be involved in this process. This is suggested by the observed thicker myelin sheaths upon optogenetic stimulation of the premotor cortex, concomitant with improved motor function of the corresponding limb (Bechler & Ffrench‐Constant, 2014; Gibson et al., 2014).

Based on (a) the metabolic challenge endured by myelinating cells during development (Forbes & Gallo, 2017; McKenzie et al., 2014); (b) the requirement of a regular supply of myelin components in adult life (in homeostasis and during adaptive myelination) (Bechler & Ffrench‐Constant, 2014; Gibson et al., 2014); and (c) the central role of remyelination after injury and in disease (Cole, Early, & Lyons, 2017), signaling pathways central to cell metabolism are likely to occupy strategically relevant positions in the regulation of myelination in health and disease. Among such pathways, we will focus in this review mainly on the role of mTOR and closely associated signaling components.

## 2 mTOR AND METABOLISM

**Figure 2:**
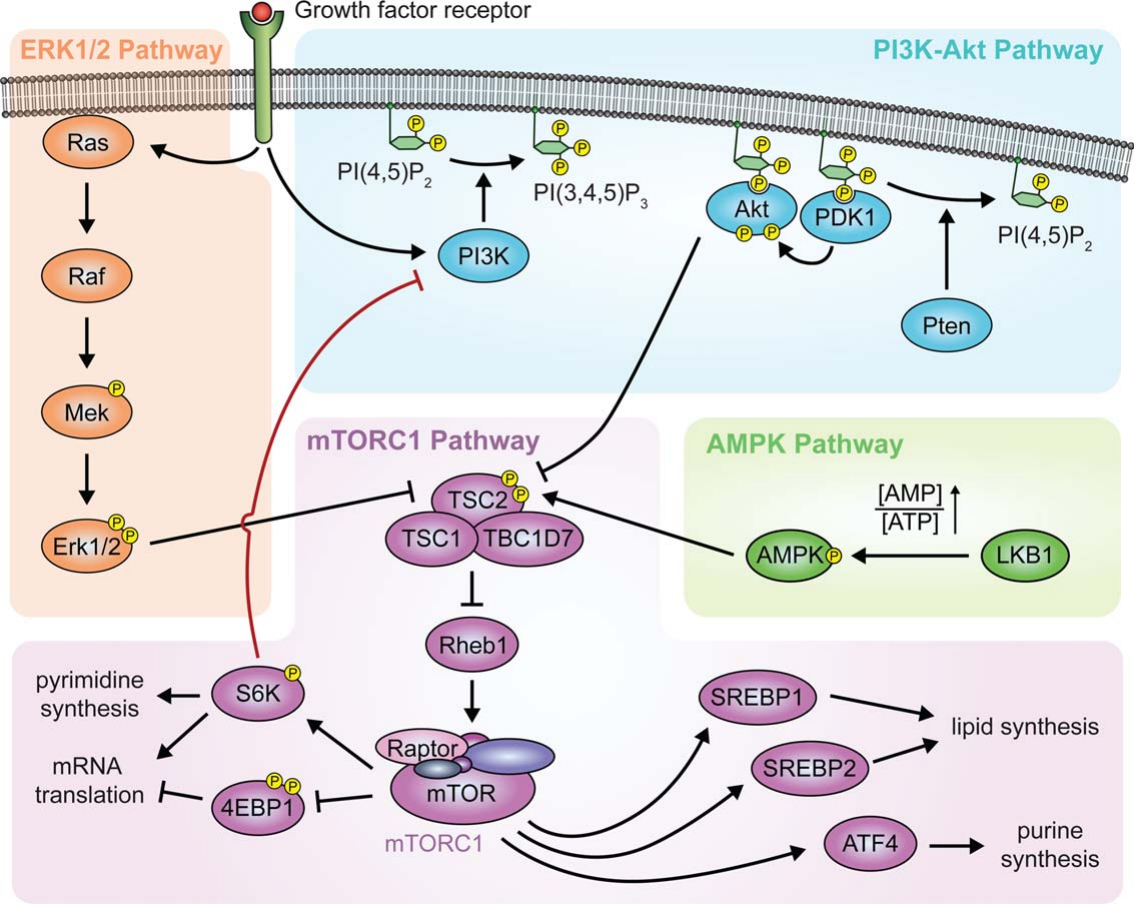
mTORC1 and its upstream pathways. A schematic representation of the main components of the mTORC1 pathway and major upstream pathways controlling mTORC1 activity is shown. The red line highlights the major inhibitory feedback loop from mTORC1 to PI3K‐Akt

The mechanistic Target Of Rapamycin (mTOR), a major gatekeeper of anabolism, is a protein serine/threonine kinase giving rise to two complexes with different sensitivity to rapamycin (Saxton & Sabatini, 2017): mTOR complex 1 (mTORC1) is sensitive and mTOR complex 2 (mTORC2) is resistant to this drug, although prolonged treatment was reported to inhibit also the latter (Lamming et al., 2012; Sarbassov et al., 2006). While the functions of mTORC2 are rather elusive, it is firmly established that mTORC1 is a central coordinator of virtually every aspect of cell growth (Figure 2).

### 2.1 mTORC1, a central coordinator of metabolism

A main functional role of mTORC1 is the regulation of mRNA translation via its targets 4EBPs and S6Ks. Unphosphorylated 4EBP1 binds to and sequesters eIF4E away from the cap‐binding complex, resulting in a block of cap‐dependent mRNA translation (Thoreen, 2017). mTORC1 counteracts these events by phosphorylating 4EBP1 to release eIF4E and, consequently, promote translation. Although the molecular details are not completely understood, this mechanism seems to increase particularly the translation of mRNAs with a pyrimidine‐rich stretch in their 5′UTRs, called TOP (terminal oligopyrimidine) or TOP‐like motifs (Thoreen et al., 2012). Additionally, S6K1 promotes the helicase activity of eIF4A, which is assumed to enhance translation by unwinding long and complex 5′UTRs (Ma & Blenis, 2009).

Another well‐established function of mTORC1 is the promotion of lipid synthesis. The SREBP transcription factors (SREBP1a, SREBP1c, and SREBP2) are fundamental mediators of this function (Jeon & Osborne, 2012). mTORC1 promotes the processing of SREBPs to their mature forms. Subsequently, mature SREBPs induce the expression of several enzymes involved in fatty acid and cholesterol synthesis (Porstmann et al., 2008). Although the exact mechanisms are not yet defined, S6K1 has been implicated in this process (Düvel et al., 2010; Owen et al., 2012). In addition, mTORC1 was shown to modulate the actions of mature SREBPs by regulating the localization of lipin‐1. When mTORC1 is suppressed, lipin‐1 translocates to the nucleus and inhibits nuclear accumulation of SREBP1 and SREBP2 (Peterson et al., 2011).

Recent studies have expanded the spectrum of anabolic reactions that are regulated by mTORC1, revealing that pyrimidine and purine de novo synthesis is also promoted downstream of mTORC1. In the first case, S6K1 phosphorylates the enzyme CAD to increase the production of *N*‐carbamoyl‐l‐aspartate, a pyrimidine precursor (Ben‐Sahra, Howell, Asara, & Manning, 2013). In the second case, mTORC1 promotes the translation of the transcription factor Atf4, which in turn induces the transcription of Mthfd2, an enzyme in the purine pathway (Ben‐Sahra, Hoxhaj, Ricoult, Asara, & Manning, 2016).

### 2.2 From growth factors to mTORC1 and back

In multicellular organisms, the growth of individual cells needs to be coordinated with the growth of the entire tissue and organism. Consistent with this requirement, in addition to its regulation by nutrient availability such as amino acids and cholesterol (Castellano et al., 2017; Wolfson & Sabatini, 2017), the mTORC1 pathway in metazoans is tightly wired to growth factor signaling. The TSC complex, formed by the subunits TSC1, TSC2, and TBC1D7, is responsible for relaying most growth factor signaling pathways to mTORC1 (Ben‐Sahra & Manning, 2017). The TSC complex acts as a GTPase‐activating protein (GAP) complex for the small GTPase Rheb1, a potent mTORC1 activator when loaded with GTP (Patel et al., 2003; Saucedo et al., 2003; Stocker et al., 2003). In the absence of growth factor stimulation, the TSC complex is active and reduces the levels of GTP‐loaded Rheb1. Consequently, mTORC1 activity is inhibited. In contrast, when growth factors are present, the GAP activity of the TSC complex is suppressed and mTORC1 is activated.

Suppression or promotion of the GAP activity of the TSC complex depends on protein phosphorylations, mainly downstream of growth factor receptors. Akt and Erk1/2 phosphorylate TSC2 to inhibit the TSC complex by disrupting the association between TSC1 and TSC2 (Ma, Chen, Erdjument‐Bromage, Tempst, & Pandolfi, 2005; Menon et al., 2014). In contrast, under stress conditions such as energy depletion resulting in an increased AMP to ATP ratio, the protein kinase AMPK is activated through phosphorylation by upstream kinases, such as LKB1 (Hardie, Ross, & Hawley, 2012), and phosphorylates TSC2 to enhance its GAP activity (Inoki, Zhu, & Guan, 2003). Thus, since the TSC complex integrates multiple upstream signals to negatively regulate a key step in mTORC1 activation, its disruption causes constitutive mTORC1 activation (Byles et al., 2013; Castets et al., 2013; Kwiatkowski et al., 2002).

In turn, inhibitory feedback loops initiated by mTORC1 dampen the activation of growth factor signaling pathways. Such mechanisms are well established for the PI3K‐Akt pathway and evidence indicates that also the Mek‐Erk1/2 pathway is subject to analogous feedback inhibition (Carracedo et al., 2008). Among the inhibitory feedback loops, arguably the best characterized involves S6K‐dependent phosphorylation and degradation of IRS1, a large protein adaptor that recruits PI3K and components of other signaling pathways downstream of insulin or IGF receptors (Harrington et al., 2004; Haruta et al., 2000; Shaw, 2011; Takano et al., 2001).

## 3 MYELINATION AND mTOR

**Figure 3:**
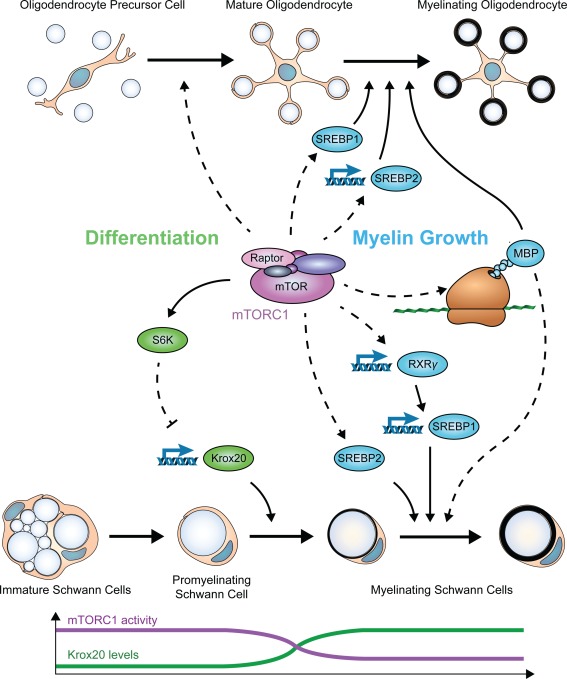
Molecular events downstream of mTORC1 during cell differentiation and myelin growth. Before onset of myelination, mTORC1 controls differentiation of myelinating cells. In the PNS, it suppresses transiently Krox20 expression via S6K and thus the transition from promyelinating to myelinating SCs. A decline in mTORC1 activity releases this block and allows myelination to proceed. In the CNS, mTORC1 activity promotes differentiation of OLs from OPCs through unknown mechanisms. After onset of myelination, mTORC1 positively regulates myelin production in both the PNS and CNS. In the PNS, mTORC1 signaling increases expression of SREBP1c via the transcription factor RXRγ, while probably promoting SREBP2 activation through post‐translational mechanisms. In the CNS, mTORC1 positively regulates at the transcriptional level expression of SREBP2, but not SREBP1c. Additionally, mTORC1 signaling stimulates translation of MBP. How mTORC1 activity changes during development of SCs with respect to Krox20 levels is graphically indicated in the bottom part of the figure. No analogous information is yet available for OL‐lineage cell development. Dashed lines indicate indirect and/or in detail unknown mechanisms

Consistent with the metabolic challenge posed by myelination, mTORC1 is indispensable for this process. Yet the role of mTORC1 in myelinating cells is more complex than mere coordination of myelin synthesis (Figure 3).

### 3.1 From cell differentiation to myelin production

Multiple loss‐of‐function approaches with transgenic mice have been employed to investigate the function of the mTOR complexes in myelinating cells. Ablation of Raptor or Rheb1 was used to suppress mTORC1 function (Bercury et al., 2014; Lebrun‐Julien et al., 2014; Norrmén et al., 2014; Zou et al., 2014), ablation of Rictor to suppress mTORC2 function (Bercury et al., 2014; Lebrun‐Julien et al., 2014; Norrmén et al., 2014), and ablation of mTOR itself to disrupt the functions of both complexes (Sherman et al., 2012; Wahl, McLane, Bercury, Macklin, & Wood, 2014). In each case, persistent hypomyelination was observed only in the absence of mTORC1 function, alone or in combination with loss of mTORC2. Nevertheless, loss of only mTORC2 function had detectable consequences in the CNS. Deletion of Rictor in OL‐lineage cells caused a transient hypomyelination (Lebrun‐Julien et al., 2014) and transiently decreased expression of various myelin proteins (Bercury et al., 2014). Moreover, when both mTORC1 and mTORC2 functions were lost, more pronounced hypomyelination and molecular changes followed as compared with single disruption of mTORC1 (Lebrun‐Julien et al., 2014).

A puzzling observation was that certain CNS regions seem to tolerate the absence of mTORC1 signaling. Deletion of Raptor, Rheb1, or mTOR in OL‐lineage cells caused hypomyelination in the spinal cord and cerebellum, but not in the brain and optic nerve (Bercury et al., 2014; Lebrun‐Julien et al., 2014; Wahl et al., 2014; Zou et al., 2014). It was proposed that an increase in Mek‐Erk1/2 signaling selectively in the corpus callosum of Raptor mutants may compensate for the loss of mTORC1 function (Bercury et al., 2014). Alternatively, the differences could depend on the Cre lines that were used to drive recombination and loss of proteins in these studies. Different from the results obtained with CNPCre, conditional ablation of Rheb1 using Olig1Cre or Olig2Cre impaired also brain and optic nerve myelination (Zou et al., 2014). Because expression of the latter Cre transgenes is reported to be active earlier than CNPCre (Zou et al., 2014), a conceivable hypothesis is that some brain OL‐lineage cells may require mTORC1 mainly in a narrow temporal window. Analogous considerations concerning Cre‐driver lines should be kept in mind also when interpreting the loss‐of‐mTORC2 function results described above.

Which mTORC1 functions are relevant for myelination? Similar to its role in other cell types, mTORC1 in myelinating cells is likely to coordinate protein and lipid synthesis for myelin production. Indeed, disruption of mTORC1 affected the levels of various myelin proteins. In particular, MBP was strikingly reduced without major changes in its mRNA level (Bercury et al., 2014; Lebrun‐Julien et al., 2014), indicating that translation of this and potentially other myelin proteins is specifically promoted by mTORC1. However, additional experimental confirmations are needed, together with insights into the underlying mechanisms. Because MBP mRNA is delivered to oligodendroglial processes during myelination and translated locally (Muller, Bauer, Schafer, & White, 2013), it will also be important to explore how mTORC1‐dependent regulation integrates with the need to spatially and temporally control MBP translation.

Myelin lipids were also reduced upon deletion of Raptor (Lebrun‐Julien et al., 2014; Norrmén et al., 2014). This correlated with reduced expression or activation of the mTORC1‐regulated transcription factors SREBP1c and SREBP2. Specifically, SREBP1c mRNA levels were decreased in the PNS, while SREBP2 mRNA levels were decreased only in the CNS. In SCs, but not in OLs, the transcription factor RXRγ was shown to be transcriptionally regulated by mTORC1 and to control, in turn, the transcription of SREBP1c downstream of mTORC1 (Lebrun‐Julien et al., 2014; Norrmén et al., 2014). Differently from other cell types (Düvel et al., 2010; Owen et al., 2012), the mTORC1 target S6K is probably not majorly involved in regulating SREBPs. Thus, myelinating cells seem to possess specific mechanisms to regulate SREBPs and the expression of enzymes for lipid synthesis, with the additional twist that these mechanisms are probably different between SCs and OLs. Although overshadowed by the role of mTORC1, also mTORC2 has been implicated in lipid metabolism (Laplante & Sabatini, 2009). This could partially explain the stronger defects observed in OLs‐Raptor/Rictor double mutants (Lebrun‐Julien et al., 2014). Intriguingly, at least in yeast, mTORC2 has been already linked to the biosynthesis of sphingolipids (Aronova et al., 2008), a lipid class highly represented in myelin (Chrast, Saher, Nave, & Verheijen, 2011).

Besides its role in coordinating myelin synthesis, additional essential functions of mTORC1 before myelination onset have emerged. mTORC1 was shown to promote differentiation of OLs from OPCs. In the absence of Rheb1 or Raptor, a reduction in OLs and an accumulation of OPCs were detected (Bercury et al., 2014; Zou et al., 2014), in line with a previous report in which rapamycin inhibited the progression of O4‐positive late OPCs to GalC‐positive immature OLs in vitro (Tyler et al., 2009). Besides its direct regulation of metabolic reactions, mTORC1 has a profound impact on the cell transcriptome (Düvel et al., 2010) and controls several transcription factors directly or via its downstream targets. Thus, it is possible that also one or more transcription factors with roles in OL differentiation are acting downstream of mTORC1. For instance, Id2 and Id4, known to inhibit OL differentiation, were reported to be upregulated after rapamycin treatment (Tyler et al., 2009).

In contrast to its positive role in promotion of myelin growth, in PNS development high mTORC1 activity inhibits SC differentiation from promyelinating (i.e., SC in contact with a single axon, ready to start myelination) to myelinating cells, as indicated by the following experimental evidence. First, under normal physiological conditions, mTORC1 activity in SCs is high prior to the onset of myelination and declines to lower levels as cells progress from the promyelinating stage further (Beirowski, Wong, Babetto, & Milbrandt, 2017; Figlia, Norrmen, Pereira, Gerber, & Suter, 2017; Heller et al., 2014). Second, mTORC1 hyperactivation due to SC‐specific deletion of TSC1 or the negative regulator of PI3K‐Akt signaling PTEN delayed onset of myelination (Beirowski et al., 2017; Figlia et al., 2017), while even higher mTORC1 activity following deletion of TSC2 persistently arrested onset of myelination (Beirowski et al., 2017). The physiological decline in mTORC1 activity is therefore required to allow onset of SC myelination. On the molecular level, aberrantly high mTORC1 signaling impaired the timely upregulation of Krox20 (Egr2) expression (Figlia et al., 2017), the master transcription factor required for the onset of PNS myelination (Svaren & Meijer, 2008; Topilko et al., 1994). Downstream of mTORC1, S6K appears to be the responsible mediator by inhibiting transcriptionally Krox20 expression. Consistent with these findings, Krox20 levels were conversely upregulated in SC‐specific Raptor mutants and in SCs treated with rapamycin in vitro (Figlia et al., 2017). The physiologically high mTORC1 activity before onset of myelination is likely to contribute to a transient halt of the SC differentiation program to allow completion of radial sorting of large axons by immature SCs before starting the process of myelination. Such a mechanism is consistent with the findings that radial sorting was accelerated in the presence of high mTORC1 activity, but impaired in the opposite case (Figlia et al., 2017; Norrmén et al., 2014). In addition, direct or indirect promotion of SC proliferation by high mTORC1 activity might contribute as a causative differentiation‐inhibitory factor (Beirowski et al., 2017; Figlia et al., 2017).

With better definitions of the roles of mTORC1 in myelinating cells, also the therapeutic potential of manipulating its activity has become clearer. Inhibition of mTORC1 with rapamycin or improved analogues, an approach already used successfully in cardiology and transplantation medicine (Benjamin, Colombi, Moroni, & Hall, 2011), could curb excessive myelin growth and myelin aberrations that occur in a number of hereditary neuropathies (Suter & Scherer, 2003). Indeed, rapamycin treatment was able to correct exuberant myelin growth in a mouse model of tomaculous neuropathy (Goebbels et al., 2012). A reduction in mTORC1 activity may also underlie the proposed beneficial effect of inhibiting neuregulin‐1 (NRG1) type III signaling in similar neuropathy models (Bolino et al., 2016), although the role of NRG1 signaling in neuropathies is complex (Fledrich et al., 2014). In light of the novel data indicating a prodifferentiating effect of mTORC1 inhibition in SCs, rapamycin treatment may also be a therapeutic option in neuropathies in which SC differentiation is defective, often a clinically severe condition. Intriguingly, administration of rapamycin has already been shown to decrease p75 expression levels, a marker for immature and promyelinating SCs, and to increase myelin proteins and myelinated fibers in a mouse model of hereditary neuropathy (Nicks et al., 2014). Such a therapeutic approach would be potentially favored by the intrinsic biological plasticity of SCs, which—unlike OLs—are capable of dedifferentiation/demyelination and redifferentiation/remyelination (Kim, Mindos, & Parkinson, 2013). In this respect, it is striking that treatment with rapamycin led to the resumption of the SC myelination program and behavioral improvements in an animal model with chronic arrest of SCs at the promyelinating stage due to deletion of TSC2 (Beirowski et al., 2017).

Conversely, therapeutic strategies aimed at increasing mTORC1 activity might be considered in various diseases with hypomyelination and after injury, both in the CNS and PNS. However, there are caveats with this approach, as we will discuss next.

### 3.2 More is less

The outcome of mTORC1 hyperactivation in OLs, after disruption of the TSC complex by deleting TSC1, is hypomyelination (and not the expected hypermyelination) (Jiang et al., 2016; Lebrun‐Julien et al., 2014). Hypomyelination and a reduction in OL‐lineage cells were also reported after deletion of TSC2 (Carson et al., 2015). These results are in line with reports of abnormal white matter microstructure in the brain of patients with tuberous sclerosis, a systemic disorder caused by loss‐of‐function mutations of TSC1 or TSC2 (Ercan et al., 2017). Additionally, after the delayed onset of myelination upon deletion of TSC1 in the PNS as discussed above, mutant SCs produced thinner myelin sheaths (Figlia et al., 2017). At least five mechanisms might account for these paradoxical results.

First, radial hypomyelination in the PNS may directly derive from the delayed onset of myelination caused by early mTORC1 hyperactivation. Consistent with this hypothesis, inducible deletion of TSC1 in SCs already committed to myelination resulted in radial hypermyelination (Figlia et al., 2017), in contrast to the differentiation‐inhibitory effects of high mTORC1 activity before myelination onset.

Second, hyperactivation of mTORC1 could trigger nonphysiological toxic effects, for instance ER stress and unfolded protein response (UPR) (Ozcan et al., 2008). Indeed, deletion of TSC1 in OL‐lineage cells caused UPR and apoptosis of OLs, which could be partially ameliorated by a drug alleviating ER stress (Jiang et al., 2016). However, because the reversal of the phenotype was incomplete, other mechanisms might also be involved.

Third, one or more of the TSC subunits could exert noncanonical functions independent of mTORC1 signaling. However, because treatment with rapamycin was able to reverse the impaired myelination in the PNS and CNS (Beirowski et al., 2017; Carson et al., 2015; Figlia et al., 2017; Jiang et al., 2016; Lebrun‐Julien et al., 2014), this possibility seems unlikely.

Fourth, hyperactive mTORC1 could suppress mTORC2 (Xie & Proud, 2014). Although disruption of the TSC complex was indeed accompanied by suppression of mTORC2 (Beirowski et al., 2017; Figlia et al., 2017; Lebrun‐Julien et al., 2014), as indicated by lower mTORC2‐dependent phosphorylation of Akt, it is unlikely that this accounts alone for the observed hypomyelination, given the minor requirement of mTORC2 for myelination.

**Figure 4:**
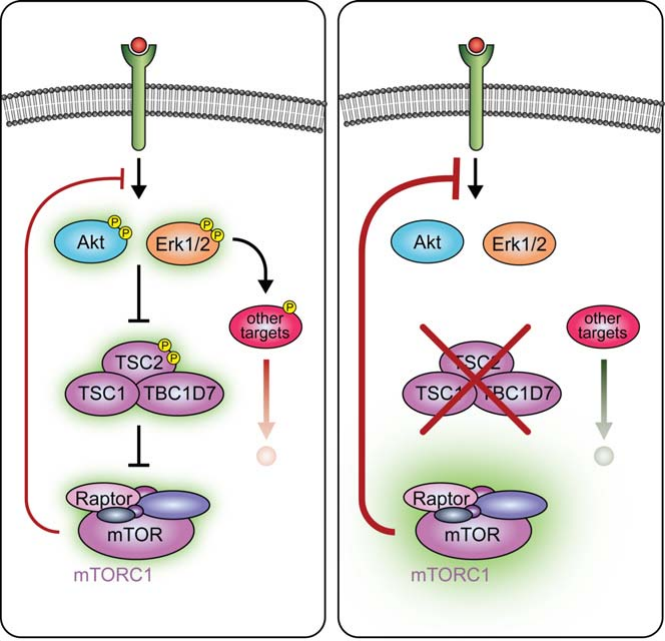
Potential perturbation of mTORC1‐independent targets upon disruption of the TSC complex. Due to feedback inhibition of the upstream pathways, hyperactivation of mTORC1 after disruption of the TSC complex (on the right) has the potential to perturb also mTORC1‐independent targets of Akt and Erk1/2 (“other targets”) leading to different outcomes (symbolized by differently colored arrows). A similar general mechanism appears to underlie paradoxical effects of TSC1 or TSC2 deletion in other cell types. The green halo indicates level of activity. Note that phosphorylation of mTORC1‐independent targets by Akt or Erk1/2 may be either activating or inhibitory

Fifth, hypomyelination due to mTORC1 hyperactivation could derive from feedback suppression of the Mek‐Erk1/2 and/or PI3K‐Akt pathways, both known to have major functions in myelination. Such mechanisms have been shown to be responsible for similar paradoxical findings upon disruption of the TSC complex in other cell types by affecting mTORC1‐independent targets of these pathways (Byles et al., 2013; Tang et al., 2014; Yecies et al., 2011) (Figure 4). Indeed, similar to other cell types, hyperactivation of mTORC1 after deletion of TSC1 or TSC2 suppressed Akt activity in both SCs and OLs (Beirowski et al., 2017; Figlia et al., 2017; Lebrun‐Julien et al., 2014). Although no changes in Mek‐Erk1/2 signaling were detected in this context, inhibition of mTORC1 was reported to increase Erk1/2 phosphorylation in the corpus callosum (Bercury et al., 2014), raising the possibility that an analogous inhibitory feedback loop from mTORC1 on Erk1/2 phosphorylation could be active in some OL subsets. Thus, the paradoxical hypomyelination in TSC1 or TSC2 mutants could also originate in part from perturbations of mTORC1‐independent targets of these two pathways upstream of mTORC1. Evidence in support of this possibility and potential implications for the regulation of myelination will be discussed next (note that we use the term “mTORC1‐independent” targets to indicate other targets than those directly affected via the mTORC1 pathway, but downstream of Akt or Erk1/2).

### 3.3 Not all roads may lead to mTORC1

**Figure 5:**
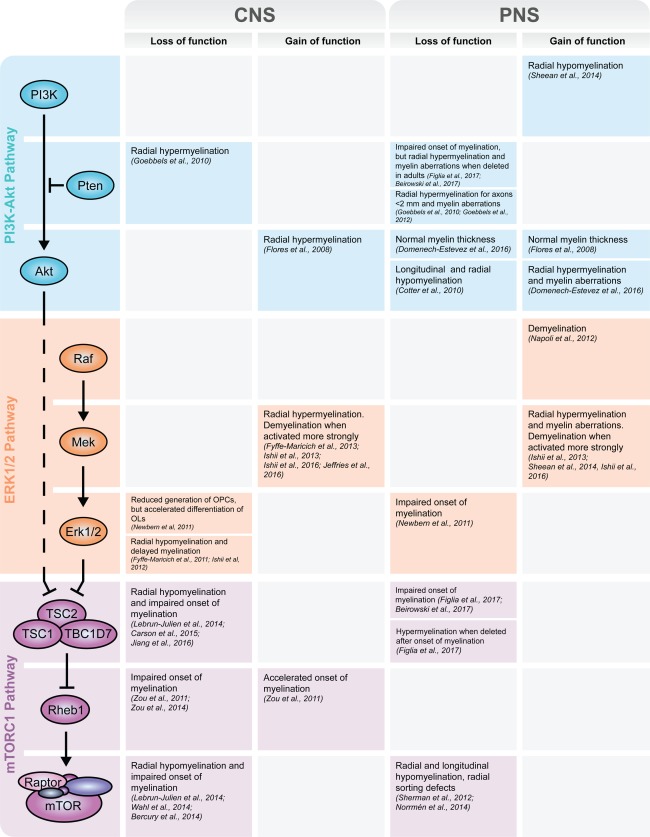
Overview of the outcomes of loss‐ or gain‐of‐function studies of various components of mTORC1 and upstream pathways. The major outcomes of the loss‐ and gain‐of‐function studies on the roles of mTORC1 and the upstream PI3K‐Akt and Mek‐Erk1/2 pathways in PNS and CNS myelination are summarized (Beirowski et al., 2017; Bercury et al., 2014; Carson et al., 2015; Cotter et al., 2010; Domenech‐Estevez et al., 2016; Figlia et al., 2017; Flores et al., 2008; Fyffe‐Maricich, Karlo, Landreth, & Miller, 2011; Fyffe‐Maricich, Schott, Karl, Krasno, & Miller, 2013; Goebbels et al., 2010, 2012; Ishii, Furusho, & Bansal, 2013; Ishii, Furusho, Dupree, & Bansal, 2016; Jeffries et al., 2016; Jiang et al., 2016; Lebrun‐Julien et al., 2014; Napoli et al., 2012; Newbern et al., 2011; Norrmén et al., 2014; Sheean et al., 2014; Sherman et al., 2012; Wahl et al., 2014; Zou et al., 2011, 2014)

PI3K controls 50–100 targets, including Akt, which in turn potentially phosphorylates more than 100 substrates (Vanhaesebroeck, Stephens, & Hawkins, 2012). Similarly, Mek‐Erk1/2 signaling is estimated to control at least 50 cytosolic targets and various transcription factors (Anjum & Blenis, 2008). It appears then plausible that one or more of these targets may act independently of mTORC1 and significantly influence myelination. A side‐by‐side comparison of studies assessing the roles of mTORC1 and known upstream pathways in myelination provides suggestive evidence in this direction (Figure 5).

In contrast to inhibition of myelination onset in not‐yet‐myelinating SCs, inducible deletion of TSC1 or PTEN in adult differentiated SCs reactivated myelin growth and resulted in radial hypermyelination (Figlia et al., 2017). However, subtle but significant differences were observed depending on the activation status of PI3K‐Akt. Specifically, radial hypermyelination progressed over time in PTEN‐deficient nerves (i.e., hyperactivation of both PI3K‐Akt and mTORC1), while it remained stable in TSC1‐deficient nerves (i.e., hyperactivation of mTORC1, but suppression of PI3K‐Akt). These results suggest that mTORC1‐independent targets of Akt may be relevant after onset of myelination to promote myelin growth. What is the identity of these targets and of the biological processes they control? In most reports on constitutive activation of PI3K and/or Akt in SCs, hyperwrapping of nonmyelinating SCs around axons in Remak bundles was a peculiar feature (Domenech‐Estevez et al., 2016; Figlia et al., 2017; Goebbels et al., 2010). This phenotype is likely independent of mTORC1, as treatment with rapamycin was not able to revert it (Domenech‐Estevez et al., 2016), and such alterations were not observed after deletion of TSC1 (Figlia et al., 2017). Thus, one possibility is that, in analogy to axonal hyperwrapping in Remak bundles, PI3K‐Akt‐dependent and mTORC1‐independent mechanisms promote wrapping of SCs around axons during developmental myelination, possibly by regulating cytoskeletal dynamics. In line with this possibility, constitutive activation of Akt was shown to induce Rac1 activity in cultured SCs (Domenech‐Estevez et al., 2016). Thus, PI3K‐Akt and mTORC1 may act synergistically, the former providing signaling for the “wrapping force” and the latter for myelin protein and lipid synthesis. mTORC1‐independent targets of PI3K‐Akt could have important functions also during OL myelination. While deletion of TSC1 or TSC2 in OLs (i.e., mTORC1 hyperactivation, but suppression of PI3K‐Akt) resulted in CNS hypomyelination and OL death (Jiang et al., 2016), expression of constitutively active Akt, or deletion of the PI3K‐Akt inhibitor PTEN (i.e., hyperactivation of both mTORC1 and PI3K‐Akt), caused marked radial hypermyelination (Domenech‐Estevez et al., 2016; Flores et al., 2008; Goebbels et al., 2010). Since Akt can promote cell survival (Manning & Toker, 2017), Akt‐dependent but mTORC1‐independent survival pathways may be required during OL myelination. Inhibition of these pathways upon mTORC1 hyperactivation might contribute to the observed apoptosis of TSC1‐mutant OLs, in addition to ER stress (Jiang et al., 2016).

Similar to PI3K‐Akt, the Mek‐Erk1/2 pathway may involve mTORC1‐independent targets with roles in myelination. Although the interpretation of the available evidence is somewhat complex due to variable experimental strategies used, the combined findings suggest that, in contrast to hyperactivation of mTORC1 following deletion of TSC1 or TSC2 (Beirowski et al., 2017; Carson et al., 2015; Figlia et al., 2017; Jiang et al., 2016; Lebrun‐Julien et al., 2014), hyperactivation of Mek‐Erk1/2 using constitutively active Mek1 causes radial hypermyelination, both in the CNS and PNS (Fyffe‐Maricich et al., 2013; Ishii et al., 2013, 2016; Sheean et al., 2014). However, the mTORC1‐independent targets of Mek‐Erk1/2 that may be responsible for the differences observed by activation of these signaling pathways are unknown. Intriguingly, the transcription factor YY1 was shown to be activated by Mek‐dependent phosphorylation and to promote Krox20 expression (He et al., 2010). Thus, increased Krox20 expression through Mek‐dependent activation of YY1 could oppose the inhibitory effect of high mTORC1 on the expression of this crucial SC transcription factor.

Finally, not only growth factor‐activated pathways, but also metabolic regulators signaling to mTORC1—such as LKB1‐AMPK—might control mTORC1‐independent targets in myelination. Deletion of LKB1 in SCs caused delayed onset of myelination, radial hypomyelination, and axonal degeneration (Beirowski et al., 2014; Pooya et al., 2014; Shen et al., 2014). Although some features of LKB1‐deficient mice are reminiscent of those upon TSC1 or TSC2 deletion (i.e., the expected phenotype), AMPK activation was paradoxically increased in LKB1‐deficient mice, while mTORC1 activity was consequently decreased (Beirowski et al., 2014), indicating that the reported impairment in myelination and axonal degeneration may have arisen from AMPK‐ and mTORC1‐independent functions of LKB1.

### 3.4 Navigating upstream

Not all axons are myelinated and not all myelinated axons are myelinated to the same extent at a given time point. Based on these observations, instructive cues originating from axons and sensed by myelinating cells were sought and found. Remarkably, several of these mediators act, or are likely to act, via mTORC1 signaling, further attesting to the importance of this signaling hub in myelination.

In the PNS, the axonally expressed and membrane‐bound growth factor NRG1 type III and its heterodimeric receptor pair ErbB2‐ErbB3 on SCs provide the dominating instructive signal for myelination. Specifically, threshold levels of this factor instruct SCs to start myelination (Taveggia et al., 2005), and in sequence, the precise levels of NRG1 type III guide SCs to produce correct amounts of myelin (Michailov et al., 2004). Downstream of NRG1, mTORC1 activation is most likely a key event. NRG1 activates both the PI3K‐Akt and Mek‐Erk1/2 pathways, and more downstream activation of mTORC1 was also observed upon in vitro stimulation of SCs with NRG1 (Figlia et al., 2017). Furthermore, loss of NRG1 stimulation strongly reduced mTORC1 activity in SCs (Heller et al., 2014), indicating that other ligand‐receptor interactions than NRG1 signaling, such as the extracellular matrix‐binding integrins, are probably minor contributors to mTORC1 activation in SCs.

How can the same intracellular signaling axis mediate the two conceptually opposing functions previously described for mTORC1 activity, that is, inhibition of SC differentiation from promyelinating stage to myelination on one hand, and promotion of myelin growth on the other? As discussed earlier, mTORC1 activity is high in early SC development and declines as SCs commit to myelination, indicating that regulated mTORC1 activity is likely to underlie the switch from one function to the other. Since different NRG1 isoforms have been implicated in different functions and signaling in various physiological settings (Mei & Nave, 2014), different NRG1 isoforms might be involved in the regulation of the higher and lower activity of mTORC1 before and after onset of myelination, respectively. Alternatively, different expression levels of the same NRG1 isoform, modulation of NRG1 by regulated proteolysis (Taveggia, 2016), and/or crosstalk interactions with other pathways affecting NRG1 downstream signaling may lead to diminished mTORC1 activation in SCs at the onset of myelination. In this respect, it was recently proposed that signals from the basal lamina, potentially via the receptor GPR126 (Paavola, Sidik, Zuchero, Eckart, & Talbot, 2014; Petersen et al., 2015), alter SC responsiveness to NRG1 (Ghidinelli et al., 2017). Despite all these hints, the question of how mTORC1 activity is affected by which signals in the various stages of SC myelination remains largely unanswered at this time.

Unlike in PNS myelination, no single instructive signal for CNS myelination has been identified so far. Rather, CNS myelination seems to depend on an intrinsic myelination program of OLs, in combination with extrinsic signals from growth factors and from the electrical activity of axons. Differently from SCs, OLs retain the potential to produce myelin also when axons are fixed with paraformaldehyde or substituted with inert artificial fibers (Lee et al., 2012; Rosenberg, Kelland, Tokar, De la Torre, & Chan, 2008). These observations suggested a “default” myelination program, intrinsic to OLs, that does not depend on other axonal signals than a threshold of axonal caliber. Extending in this direction, spinal cord‐derived OLs seeded on nanofibers formed longer internodes than brain‐derived OLs, mirroring the in vivo observation that internodes in the spinal cord are, on average, longer than in the brain (Bechler, Byrne, & Ffrench‐Constant, 2015).

Further alongside, or on top of the OL‐intrinsic program of CNS myelination, an extrinsic program consisting of signals from axons (and potentially other sources) appears to fine‐tune OL myelination, likely to match the electrophysiological needs of certain axons. A multitude of regulatory growth factor signaling pathways have been implicated in this context. However, in contrast to the PNS, NRG1 may play a modulatory, but not a dominant role. Although NRG1 is expressed at least by some CNS axons, as are NRG1 receptors by OLs, deletion of one or the others did not significantly alter CNS myelination (Brinkmann et al., 2008), or was reported to affect myelination only in some CNS regions (Taveggia et al., 2008). However, overexpression of NRG1 type I or III resulted in radial hypermyelination and an increase in myelinated axons (Brinkmann et al., 2008), possibly related to cell culture findings showing that NRG1 treatment can enhance the myelination of active axons (Lundgaard et al., 2013).

Additional research has focused on other growth factors such as the FGF family, IGF1, BDNF, and NT3. Although deletion of each of these proteins, or their cognate receptors, impaired OL myelination to some extent (Cellerino, Carroll, Thoenen, & Barde, 1997; Furusho, Ishii, & Bansal, 2017; Joseph D'Ercole & Ye, 2008; Kahn et al., 1999; Vondran, Clinton‐Luke, Honeywell, & Dreyfus, 2010; Ye, Li, Richards, DiAugustine, & D'Ercole, 2002; Zeger et al., 2007), it is unclear whether they are both necessary and sufficient to drive OL myelination, analogous to NRG1 type III in the PNS. The precise physiological cellular sources of these growth factors and the signals controlling their production and release are currently being elucidated. Intriguingly in this context, the PI3K‐Akt‐mTORC1 axis in neurons has also been implicated in the control of myelination. Deletion of PTEN in cerebellar granule cells was shown to cause myelination of axons that are normally not myelinated, likely as the result of a combination of increased axonal size and elevated expression of BDNF, FGF1, and other promyelinating factors (Goebbels et al., 2017). In contrast, pan‐neuronal mTORC1 hyperactivation upon deletion of TSC1 caused hypomyelination (Ercan et al., 2017). The TSC1‐deficient neurons were found to secrete higher amounts of CTGF and this growth factor inhibited OL maturation in a paracrine manner. Although a direct comparative analysis with cerebellar granule cells is currently lacking, these findings may reflect intrinsic differences between neuronal populations with regard to mTORC1 signaling. Alternatively, mTORC1‐independent targets of PI3K‐Akt in neurons might contribute to these different outcomes.

Other CNS cells, including astrocytes and microglia, may also release factors that can affect myelination in physiological and/or pathological conditions (Domingues, Portugal, Socodato, & Relvas, 2016). In particular, CD11c‐positive microglial cells were described as a major source of IGF1 to support developmental OL myelination (Wlodarczyk et al., 2017). Moreover, M2‐type microglia and blood‐derived macrophages were shown to promote remyelination by secreting activin‐A, potentially contributing to mTORC1 activation in OL‐lineage cells (Miron et al., 2013). On a more speculative note, analogously to neurons, the PI3K‐Akt‐mTORC1 signaling might also be involved in the production and release of myelination‐regulating growth factors by astrocytes and microglia.

Following up on pioneering work (Barres & Raff, 1994; Demerens et al., 1996), electrical axonal activity and release of synaptic vesicles have been proposed recently as a major axonal signal modulating oligodendrogenesis, regulating the numbers and lengths of internodes generated, guiding the selection of axons to be myelinated, and adjusting the growth of myelin sheaths (Gibson et al., 2014; Hines, Ravanelli, Schwindt, Scott, & Appel, 2015; Koudelka et al., 2016; Mensch et al., 2015; Wake et al., 2015; Wake, Lee, & Fields, 2011). The molecular players underlying such activity‐dependent myelination are not univocally defined. Both ATP and glutamate are released by synaptic vesicles and regulate OL myelination (Stevens, Porta, Haak, Gallo, & Fields, 2002; Wake et al., 2011), and the relative contribution of each of the three classes of glutamate receptors—NMDA, AMPA, and metabotropic receptors (mGluRs)—is somewhat controversial (De Biase et al., 2011; Fannon, Tarmier, & Fulton, 2015; Guo et al., 2012; Kougioumtzidou et al., 2017; Lundgaard et al., 2013).

The roles of growth factors and neuronal activity are not necessarily mutually exclusive. Stimulation with NRG1 and BDNF in vitro renders OL myelination dependent on glutamate via NMDA receptors (Lundgaard et al., 2013), indicating that signals from growth factors and glutamate are integrated within OL‐lineage cells. Conversely, one or more growth factors could also be released with synaptic vesicles, analogous to BDNF in the context of synaptic plasticity (Ebert & Greenberg, 2013).

How our knowledge on mTORC1 signaling integrates with this model of intrinsic and extrinsic control of OL myelination is unclear. Basal levels of mTORC1 activity within OLs might be required for their “default” program of myelination. Additionally, intrinsic differences in basal mTORC1 activity between OL subpopulations (e.g., spinal cord vs. brain OLs) could exist and, at least in part, account for their different myelinogenic potential. In turn, the extrinsic program of myelination may involve an increase (or a decrease) in the basal mTORC1 activity of OLs. Specifically, mTORC1 could be recruited for myelination downstream of one or more of the growth factors discussed above, as the known signaling triggered by these factors in other cellular systems (or in SCs, in the case of NRG1) often involves mTORC1. Although mTORC1 activity is typically under control of growth factor receptors, also glutamate can activate the PI3K‐Akt‐mTORC1 signaling axis downstream of mGluRs during synaptic plasticity (Ebert & Greenberg, 2013). This raises the intriguing possibility that mTORC1 might be analogously activated by glutamate release during electrical activity‐dependent myelination and could be involved in the reported increase in MBP translation in OL processes upon glutamate release from axons (Wake et al., 2011). Conversely, as axonal signals with a repulsive function for OL myelination have also been identified, such as JAM2 (Redmond et al., 2016), it will be interesting to examine whether part of their downstream signaling also involves inhibition of mTORC1.

## 4 CONCLUDING REMARKS

It is now firmly established that mTORC1 occupies a central role in the cellular program of myelination. Dynamic regulation of mTORC1 signaling is required for the differentiation of myelinating cells and drives myelin growth (involving the production of lipids and, likely, proteins). However, a number of new questions have arisen, including: Which upstream receptors signal to mTORC1 in myelinating cells at which time? What is the nature and role of the discussed non‐TORC1 targets in the control of myelination? Which molecular components mediate the inhibitory feedback loops that regulate mTORC1 activity in myelinating cells? To which extent is mTORC1 involved in the regulation of the intrinsic and/or extrinsic control of OL myelination, and how? What is the role of mTORC1 activity in the various diseases affecting myelin and what is the therapeutic potential of targeting mTORC1 in this context? How are mTORC1 activity levels in remyelinating cells affecting repair after lesions (McLane et al., 2017)? In this review, we have provided a conceptual framework for future investigations addressing experimentally these and other still unclear aspects of mTORC1 signaling in the SC and OL lineages. Therapeutic strategies aimed at manipulating mTORC1 activity in myelin diseases and after injury will likely benefit from the knowledge obtained.

## Conflict of interest

The authors declare no competing financial interests.
